# Learning to Cycle: A Cross-Cultural and Cross-Generational Comparison

**DOI:** 10.3389/fpubh.2022.861390

**Published:** 2022-04-28

**Authors:** Rita Cordovil, Cristiana Mercê, Marco Branco, Frederico Lopes, David Catela, Elina Hasanen, Arto Laukkanen, Patrizia Tortella, Guido Fumagalli, Cristina Sá, Boris Jidovtseff, Linus Zeuwts, An De Meester, Farid Bardid, Ricardo Fujikawa, Sanne Veldman, Silvija Zlatar, Isaac Estevan

**Affiliations:** ^1^Centro Interdisciplinar de Estudo da Performance Humana, Faculdade de Motricidade Humana, Universidade de Lisboa, Lisbon, Portugal; ^2^Departamento de Atividade Física e Saúde, Escola Superior de Desporto de Rio Maior, Instituto Politécnico de Santarém, Rio Maior, Portugal; ^3^Departamento de Desporto e Saúde, Faculdade de Motricidade Humana, Universidade de Lisboa, Lisbon, Portugal; ^4^Centro de Investigação em Qualidade de Vida, Escola Superior de Desporto de Rio Maior, Instituto Politécnico de Santarém, Rio Maior, Portugal; ^5^Faculty of Sport and Health Sciences, University of Jyväskylä, Jyväskylä, Finland; ^6^Department of Art, Music and Movement, Faculty of Education, Free University of Bolzano, Bolzano, Italy; ^7^Department of Diagnostics and Public Health, Center for Research on Motor Development in Infancy, University of Verona, Verona, Italy; ^8^Departamento de Ciências Do Movimento Humano, Universidade Federal de São Paulo, Santos, Brazil; ^9^Research Unit for a Life-Course Perspective on Health and Education, University of Liège, Liège, Belgium; ^10^Department of Movement and Sport Sciences, Ghent University, Ghent, Belgium; ^11^Department of Physical Education, University of South Carolina, Columbia, SC, United States; ^12^School of Education, University of Strathclyde, Glasgow, United Kingdom; ^13^Real Centro Universitario Escorial Maria Cristina, Madrid College of Chiropractic, Madrid, Spain; ^14^Department of Public and Occupational Health, Amsterdam Public Health Research Institute, Amsterdam University Medical Center (Amsterdam UMC), Vrije Universiteit Amsterdam, Amsterdam, Netherlands; ^15^Health Behaviour and Chronic Diseases and Methodology, Amsterdam Public Health, Amsterdam, Netherlands; ^16^Kindergarten Matije Gupca, Zagreb, Croatia; ^17^AFIPS Research Group, RIIDASS Network, Department of Teaching of Music, Visual and Corporal Expression, University of Valencia, Valencia, Spain

**Keywords:** cycling, country, generation, active travel, children

## Abstract

**Background:**

Learning to cycle is an important milestone for children, but the popularity of cycling and the environmental factors that promote the development and practice of this foundational movement skill vary among cultures and across time. This present study aimed to investigate if country of residence and the generation in which a person was born influence the age at which people learn to cycle.

**Methods:**

Data were collected through an online survey between November 2019 and December 2020. For this study, a total of 9,589 responses were obtained for adults (self-report) and children (parental report) living in 10 countries (Portugal, Italy, Brazil, Finland, Spain, Belgium, United Kingdom, Mexico, Croatia, and the Netherlands). Participants were grouped according to their year of birth with 20-year periods approximately corresponding to 3 generations: 1960–79 (generation X; *n* = 2,214); 1980–99 (generation Y; *n* = 3,994); 2000–2019 (generation Z; *n* = 3,381).

**Results:**

A two-way ANOVA showed a significant effect of country, *F*_(9,8628)_ = 90.17, *p* < 0.001, $\eta_{\text{p}}^{2}$ = 0.086, and generation, *F*_(2,8628)_ = 47.21, *p* < 0.001, $\eta_{\text{p}}^{2}$ = 0.122, on the age at which individuals learn to cycle. Countries with the lowest learning age were the Netherlands, Finland and Belgium and countries with the highest learning age were Brazil and Mexico. Furthermore, the age at which one learns to cycle has decreased across generations. There was also a significant country x generation interaction effect on learning age, *F*_(18,8628)_ = 2.90, *p* < 0.001; however, this effect was negligible ($\eta_{\text{p}}^{2}$ = 0.006).

**Conclusions:**

These findings support the socio-ecological perspective that learning to cycle is a process affected by both proximal and distal influences, including individual, environment and time.

## Introduction

Learning to cycle is an important milestone in childhood that has important implications for future life (1, 2). Across many cultures, the use of bicycles is the first personal active traveling behavior for children to extend their territorial scope, ranging from home to school and other meaningful places (3–6). Being able to cycle extends children's possibilities to actively play outdoors and increases their autonomy, allowing for independent travel at longer distances and higher speeds than walking (7). Cycling is considered to be a foundational movement skill, since it impacts an individual's capability to be physically active and can promote physical activity and health across the lifespan (8). Moreover, cycling is associated with extensive health, economic and environmental benefits (9, 10). In some contemporary cities, as people start valuing sustainability and believing that the car's liabilities outweigh its benefits, the bicycle has emerged as an alternative to the car, mainly for commuting (11, 12). Cycling as a means to actively commute, has several positive health implications such as enhancement of cardiorespiratory fitness (13). Additionally, bicycles are a non-polluting (or low-polluting in the case of electric bicycles), highly energy-efficient mode of active and sustainable transport. As such, cycling can be considered central to different sustainable development goals outlined by the United Nations, including promoting healthy lives and well-being for all and making cities and communities inclusive, safe, resilient and sustainable (14).

Although the benefits of cycling are highly recognized, the popularity of cycling among people, as well as the status of cycling in transport infrastructure and policy, have varied across time and cultures (15–18). This has potentially influenced the importance placed on learning to cycle in different countries across different generations. The influence of time and culture in the process of learning to cycle can be framed by Bronfenbrenner's bioecological theory (19). This theory states that development occurs through an evolving process of reciprocal interactions between the person and multidimensional levels of the environment. Specifically, Bronfenbrenner's Process-Person-Context-Time (PPCT) model can be used to theoretically describe the process of learning to cycle. Learning to cycle can be seen as a proximal process that is dependent on the reciprocal interaction between the person and the environment, which occurs on a fairly regular basis over an extended period of time (19, 20). From this perspective, the individuals who teach the child to cycle as well as the bicycle itself are important elements of the immediate environment with which the child interacts regularly during the learning process.

According to the PPCT model, the process of learning to cycle will also be influenced by the personal characteristics of the child who is learning (e.g., age, skill level or motivation). Each child will learn at a different pace. This individual learning process is also impacted by the child's environment, involving four interrelated systems, from proximal to distal: (a) the microsystem, (b) the mesosystem, (c) the exosystem and (d) the macrosystem. The microsystem refers to the environments where face-to-face interactions occur, such as the child's home, school, or peer group. If people in these microsystems (e.g., parent/caregiver) value cycling and offer the child opportunities to regularly practice cycling, the learning process is enhanced (21). The mesosystem refers to the interactions between different microsystems (e.g., family- and peer relationships). The exosystem refers to the contexts that have an indirect influence on a child's development, even though the child is not an active participant in these contexts (e.g., parents' work context). Finally, the macrosystem refers to the social and cultural context in which the child is immersed, with its set of values and traditions. If, in a given culture, cycling is valued and bicycle-friendly infrastructures (e.g., bike lanes, safe paths) allowing children to cycle safely are available, it is expected that more children will use their bicycles to travel to school or to hang out with friends than when those conditions are not present (22). The final concept of the PPCT model is time. Time is related to the chronosystem, which consists of the socio-historical context the child lives in, as well as the changes that occur over the child's life course. For example, the importance given to cycling and active transportation has changed across different generations (15, 23) as well as the type of bicycles available for learning (15, 24). Even within the same generation, the average age at which children are given their first bicycle could vary and this will influence the learning process (19, 20).

The social, cultural and geographic influence on the development of foundational movement skills is also considered in the lifelong physical activity model proposed by Hulteen et al. (8). The authors suggest that foundational movement skills, such as cycling, should be viewed through a “socio-cultural and geographical” filter. That is, the importance placed on learning certain skills will vary across different cultures and geographic locations (i.e., countries, regions). As such, cycling may be considered a more important foundational skill in countries where cycling is highly valued, compared to countries where a cycling culture is less present (23, 25, 26). For instance, in the Netherlands cycling is frequently used to commute from home to school or work and learning to cycle is an important first step to maintain health-enhancing physical activity habits across the lifespan; however, in other countries (e.g., Brazil), learning how to play soccer is culturally considered as a better way to fulfill that purpose. Learning a foundational movement skill—a result of a simultaneously proximal and distal process—needs to be framed by its cultural geography. That is, where cycling is considered a socially valued travel mode, also enabled by bicycle–friendly urban planning, it can help increase the perceived importance of learning and developing such a foundational skill. As noted by Hulteen et al. (8), these cultural constraints can influence the timing and onset of cycling.

The adoption of Bronfenbrenner's bioecological theory (1995) and Hulteen and colleagues' conceptual model of physical activity across the lifespan (2018) provides a comprehensive approach to understanding the process that enables a child to learn to cycle. In the present study, we focus specifically on studying the influence of the macrosystem and the chronosystem in the learning process. More specifically, an international online survey was created to investigate the association of (1) the country of residence (macrosystem), and (2) the historical time or generation in which a person was born (chronosystem) in the age of learning how to autonomously cycle (with pedals and without training wheels). It was hypothesized that the age of learning how to cycle would differ across geographical locations of residence and generation of birth (8, 20).

## Methods

### Survey

The international project L2Cycle (Learning to Cycle) aims to assess different aspects related to the process of learning to cycle in different countries (e.g., learning age, learning paths, type of bicycles used, people involved in the learning process). For this purpose, a survey was created on LimeSurvey and it was hosted at the Faculty of Human Kinetics (University of Lisbon) server. The survey was approved by the Faculty of Human Kinetics ethics committee (process number 22/2019), launched online on November 22, 2019 and publicized through social media (Facebook, Instagram, Twitter and WhatsApp), and by email. In addition, partnerships with cycling federations, children's and parents' magazines and non-profit cycling organizations were established in different countries for dissemination on their websites and in paper magazines. Data for the current study were collected between November 22, 2019 and December 2, 2020.

At a first stage, an initial version of the survey was developed by a group of four experts in child development and was tested online among 485 participants. A sub-sample of 30 participants was additionally asked about the comprehension of the survey. Adjustments were made accordingly (e.g., deleting questions related to age of achieving certain motor milestones, and some questions were reformulated refining questions to improve clarity). At a second stage, the survey was discussed with a group of five international experts who provided further suggestions (e.g., adding questions regarding mother language and different seasons of the year). Finally, the survey was translated into 10 different languages [Portuguese (Portugal and Brazil), English, German, Croatian, Finnish, French, Dutch, Italian, Japanese and Spanish], by experts on motor behavior or motor development, and validated by country specific native speakers.

The final form of the survey took approximately 5 to 15 min to complete (depending on the number of children), could be answered anonymously, and comprised three sections:

1. “About you”-Questions about the participant's own cycle experience and biographical data (e.g., place of residence, age, sex, physical activity habits, if they know how to cycle, if not–why not, if yes-when did they learn how to cycle, what types of bicycles were used and in what sequence, where did they learn, who taught them, how often do they cycle, what do they use it for).

2. “About your oldest child” (to be completed only if the participant has at least one child)-These questions are the same as the questions in the first group but regarding the participant's oldest child.

3. “About your youngest child” (to be completed only if the participant has more than one child)-These questions are the same as the questions in the first group but regarding the participant's youngest child.

### Sample

A total of 10,640 responses regarding adults and children (parental responses) living in 29 countries were completed online. For the purpose of this study, only countries with more than 80 responses were considered. This number was based on a sample size calculation performed using the G ^*^ Power 3.1.7 Software (Universität Düsseldorf, Germany), considering the analysis of variance (Anova–one way), for a power of 0.8, alpha significance level ≤ 0.05, and estimated effect size of 0.25 (minimum difference to be detected = 2.05 and estimated SD = 10). In order to analyze differences in earliest independent cycling age across generations, participants were grouped according to their year of birth considering 20-year periods that roughly correspond to 3 generations: 1960–79 (generation X; *n* = 2,214; 1980–99 (generation Y, or the millennials; *n* = 3,994); 2000–2019 (generation Z; *n* = 3,381). Responses regarding children born in 2020 (too young to be able to ride a bicycle) or adults born before 1960 - a limited number in many countries-were not included in this study. Descriptive data of the participants included in this study is presented in Table 1.

**Table 1:** Sample distribution (*n*), means and standard deviations of age (years), sex distribution (%), and frequency of people who know how to ride a bicycle (%) in the sample of study.

**Country**	***n***				**Age (years)**	**Sex (%)**		**Able to cycle (%)**	
	**Total**	**Gen X**	**Gen Y**	**Gen Z**	**M (SD)**	**Female**	**Male**	**No**	**Yes**
Portugal	2,386	562	828	996	26.05 (15.36)	57.4	42.6	12.5	87.5
Italy	1,585	370	714	501	27.60 (14.88)	64.7	35.3	9.0	91.0
Brazil	1,455	381	650	424	29.06 (14.91)	64.2	35.8	13.1	86.9
Finland	906	240	367	299	28.82 (16.00)	56.0	44.0	5.0	95.0
Spain	884	153	381	350	25.77 (14.77)	49.4	50.6	9.3	90.7
Belgium	703	240	223	340	24.40 (16.15)	56.8	43.2	7.8	92.2
UK	630	239	265	126	34.17 (15.03)	49.4	50.6	3.5	96.5
Mexico	552	43	318	191	23.69 (8.72)	62.4	37.6	12.1	87.9
Croatia	369	71	180	118	26.31 (14.12)	65.3	34.7	7.0	93.0
Netherlands	119	15	68	36	27.38 (13.01)	68.9	31.1	2.5	97.5
Total	9,589	2,214	3,994	3,381	27.30 (15.07)	58.9	41.1	9.7	90.3

### Statistical Analysis

Descriptive statistics and frequency analysis were used to characterize the final sample. For the subsequent analysis, a two-way ANOVA was performed to investigate the effects of country of residence (ten countries), generation (three generations), and the interaction between country and generation, on the age at which individuals learned to cycle independently (i.e., riding a traditional bicycle with pedals and without training wheels). Due to non-homogeneity of variances, unequal N HSD *post hoc* tests were used to further investigate significant interaction and main effects. The significance level was set at 0.05.

## Results

### Learning Age Across Countries

**Figure 1:**
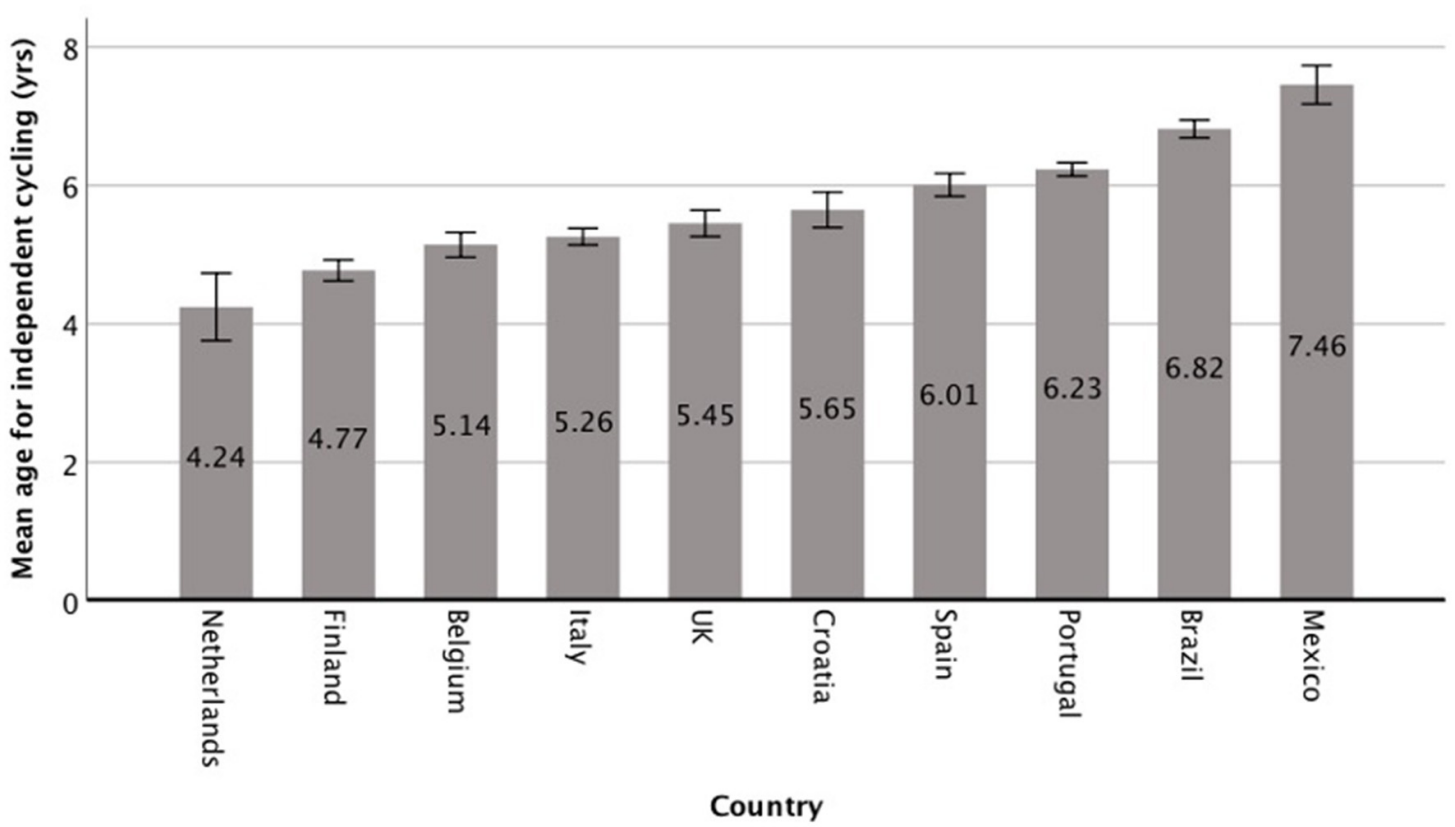
Mean age to learn how to cycle independently by country. Error bars represent 95% CI.

The country of residence significantly influences the age at which one learns how to cycle independently with pedals and without training wheels, *F*_(9,8628)_ = 90.17, *p* < 0.001, $\eta_{\text{p}}^{2}$ = 0.086. Significant differences were found in learning age among most countries (Figure 1). More specifically, our findings show different “learning age-geographical” landscapes.

In the Netherlands and Finland, learning age was significantly lower than in all other countries, except for Belgium (no significant differences). In Belgium, learning age was significantly lower than in the other countries, except for the Netherlands, Finland and Italy (no significant differences). In Italy, learning age was significantly higher than in the Netherlands and Finland, and significantly lower than in all other countries; it was not significantly different than in Belgium, UK and Croatia. In the UK, learning age was significantly higher than in the Netherlands, Finland and Belgium, and significantly lower than in Portugal, Brazil and Mexico, but not significantly different than in Italy, Spain and Croatia. In Croatia, learning age was significantly higher than in the Netherlands, Finland and Belgium, and significantly lower than in Brazil and Mexico, but not significantly different than in Italy, UK, Spain and Portugal. In Spain, learning age was significantly higher than in the Netherlands, Finland, Belgium and Italy, and significantly lower than in Brazil and Mexico, but not significantly different than in the UK, Croatia and Portugal. In Portugal learning age was significantly higher than in the Netherlands, Finland, Belgium, Italy and UK, and significantly lower than in Brazil and Mexico, but not significantly different than in Croatia and Spain. In Brazil and Mexico, learning age was significantly higher than in all other countries.

### Learning Age Over Generations

**Figure 2:**
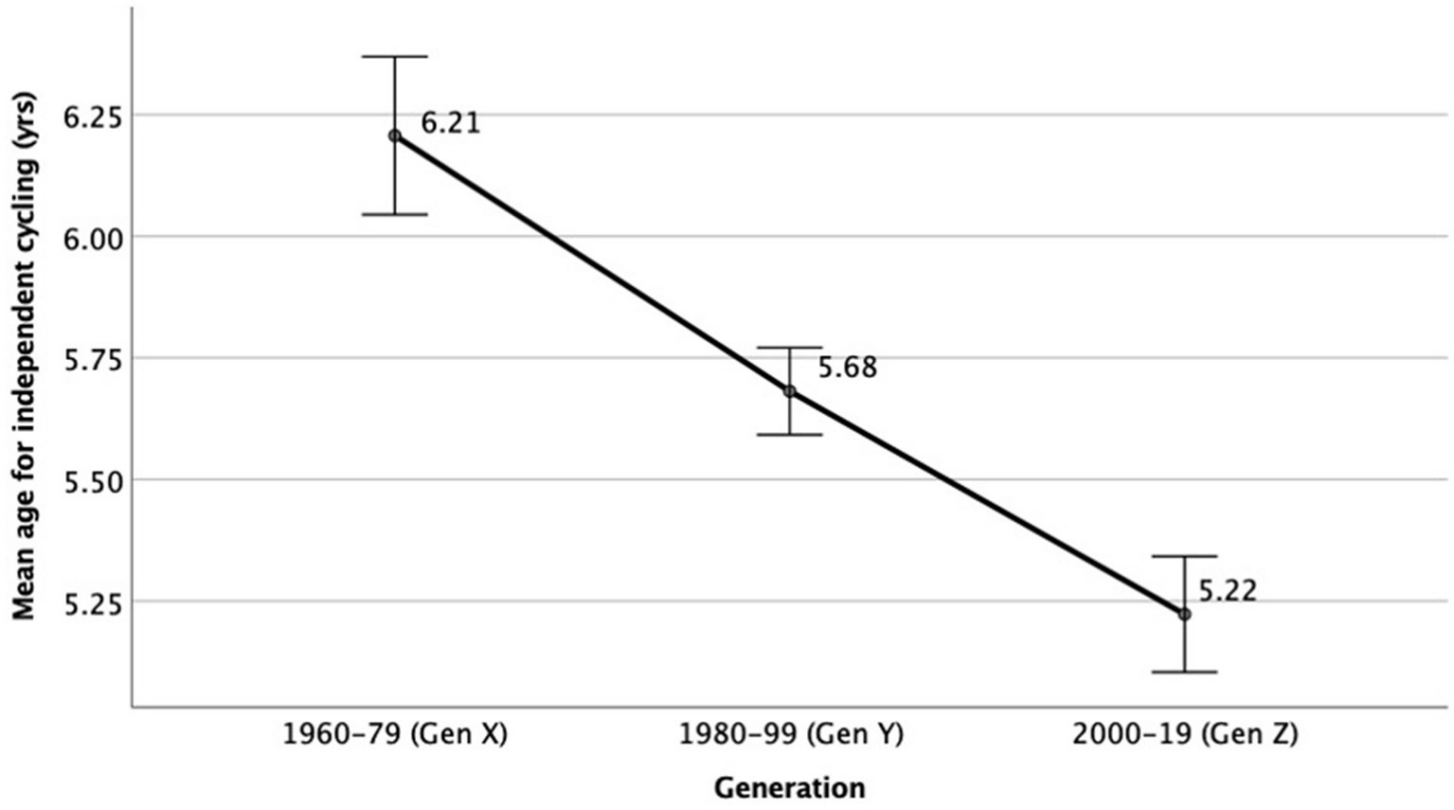
Mean age to learn how to cycle independently by generation. Error bars represent 95% CI.

There was a significant main effect of generation on the age at which a person learns to cycle, *F*_(2,8,628)_ = 47.21, *p* < 0.001, $\eta_{\text{p}}^{2}$ = 0.122. As shown in Figure 2, the learning age was lower in younger generations. *Post hoc* analysis showed there were significant differences in learning age between all generations. Specifically, Generation X (i.e., born in 1960–79) learned to cycle independently at a later age compared to Generation Y (i.e., born in 1980–99) (*p* < 0.001), as did Generation Y compared to Generation Z (i.e., born in 2000-19) (*p* < 0.001).

### Learning Age by Generation and Country

**Figure 3:**
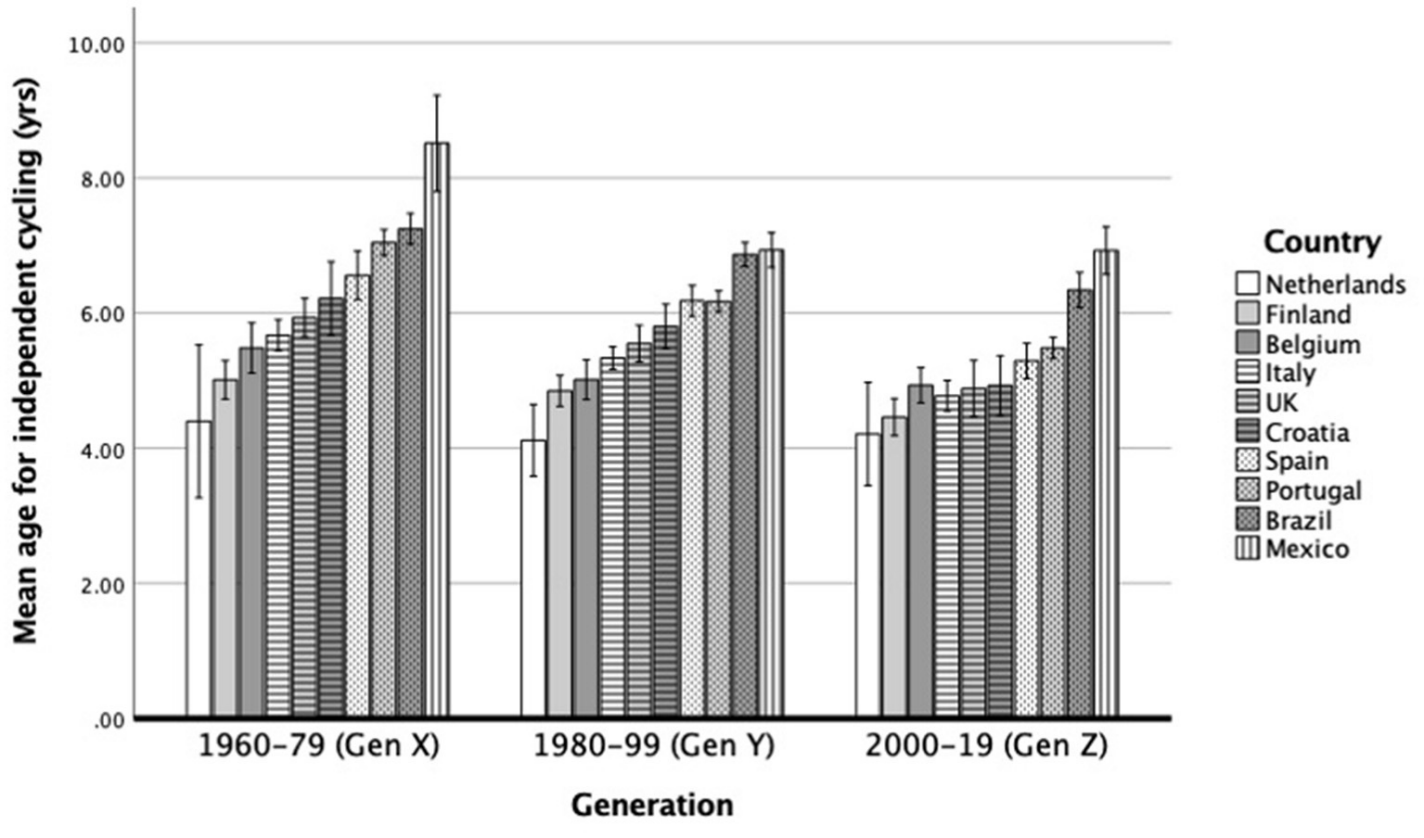
Mean age to learn how to cycle independently by generation and country. Error bars represent 95% CI.

There was a significant generation x country interaction effect on learning age, *F*_(18,8,628)_ = 2.90, *p* < 0.001. Generational changes differed slightly among countries with the decline in learning age being less evident in countries in which the learning age has been consistently low across generations (e.g., the Netherlands; see Figure 3), but the interaction effect size was negligible ($\eta_{\text{p}}^{2}$ = 0.006). The learning age for independent cycling is generally lower in later generations in all countries, and countries generally maintained their rank relative to the others across time.

## Discussion

In the present study we confirmed the hypothesized influence that the macrosystem and the chronosystem have on the age of learning to cycle (20). Results indicated that age to learn how to cycle differs across geographical locations of residence and the generation of birth, as discussed in greater detail in the next sections.

### Learning Age in Different Countries

Based on the results of the present study, we can divide the participating countries in three main groups according to the age at which children learn to cycle independently: group 1 (the Netherlands, Finland and Belgium), group 2 (Italy, UK, Croatia, Spain and Portugal), and Group 3 (Brazil and Mexico).

These group differences suggest there are geographical, social, cultural and political aspects underlying the status of active travel and cycling in different countries. These aspects are linked to spatial properties and features within the built environment, which make the places for children to learn how to cycle more or less inviting (27). The groups and the order of the countries within each group in our study also seem to reflect the cycling modal share in each country. European reports and statistics (28, 29) indicate that within Europe, the share of cycling as a travel mode varies between 27.0% in the Netherlands and 0.5% in Portugal. The order of the European countries in the cycling modal share statistics is the same as in our results (except for Croatia for which there was no information on the modal share). The Latin American countries are also known to have low levels of cycling modal share (26, 30). In fact, Goel et al. (26) studied cycling behavior in 17 countries across six continents and concluded that among the studied countries, the Netherlands had the highest level of cycling modal share (26.8%) and Brazil had the lowest (0.8 %) (Mexico was not included in the study). Thus, it seems that in countries with a higher cycling modal share, children tend to learn to cycle earlier than in countries where cycling is less popular.

In group 1 (the Netherlands, Finland and Belgium), measures for active transportation and environmental sustainability seem to be more at the heart of political concerns than in the other two groups, and therefore more embedded within the socio-cultural tissue. Northern European countries seem to be leading initiatives for transition from motorized vehicle transport to active travel, e.g., Finland has set goals to increase cycling and walking by 30% in 2030 (31) and to become carbon neutral by 2035 (32). Conversely, in Southern European or Latin American countries such initiatives are only now starting to emerge, which might explain why children in these countries learn to cycle at a later age.

Among all participating countries in this study, Netherlands, Finland and Belgium have the highest gross domestic product (GDP) per capita (33). This indicates that families in these countries may be more likely to own bicycles, which in turn provides more opportunities for children to learn and practice to cycle. The Global Matrix of Physical Activity Report Cards (34, 35) show that the Netherlands and Finland perform better on active transportation compared to other countries in the present study. In the Netherlands, cycling has been embedded in the culture for over 100 years, having been promoted as a decent and safe mode of transport for 'good civilians' since early 1900 (36). Finland's active transportation among children and youth is ranked as the second highest compared to the other countries in the present study. Active transportation among children is closely linked to children's independent mobility (CIM), which is prominent in Finland (37). Furthermore, CIM and active transportation are both dependent on community and built environment (37), an issue which also separates the Netherlands and Finland from the other countries of the present study. Children and youth living in the Netherlands, Finland, and Belgium share good results regarding the opportunities for active play (34). Active play, especially if performed outdoors, is associated with greater moderate-to-vigorous physical activity and lower sedentary levels (38), thus contributing to the development of a better motor competence (39). It is also worth noting that outdoor play is common in Finnish children's daily life at home and in early childhood education settings (40), and the snowy and icy winter months offer additional stimuli for motor learning.

Belgium is the country with the highest learning age in group 1, presenting non-significant differences with Italy (i.e., the first country in group 2). Belgian respondents on average learned to cycle around the age of five. The current results, therefore, confirm the findings of previous studies regarding the development of cycling skills in Flemish children (i.e., children living in the northern region of Belgium) (41, 42). Belgian children typically master riding a bike when progressing from kindergarten to primary school, although some regional differences exist.

Results found in group 2 (Italy, the UK, Croatia, Spain and Portugal) reflect a lower level of political commitment and investment toward active transportation, environmental sustainability and child-friendly policies among these countries. A clear example related to riding a bike as a cultural and intrinsically integrated aspect of citizen's lives is active transport in urban environments, which is also linked to CIM. Findings from the international study on CIM across 16 countries (37) revealed that England occupies the 7^th^ position, while Portugal and Italy share the 14^th^ position, as the countries in Europe with lower levels of CIM licenses. Although Italy shares the 14^th^ place with Portugal in the European CIM rank, the bicycle culture seems to be more embedded in Italy where children learn to ride a bicycle earlier than in Portugal. Between 1800 and 1900 Italy was essentially an agricultural country and the most popular sport was cycling, which was considered a “symbol of progress”. Now the bicycle is the most economical way of transport. ISTAT data from 2015 (43) showed that the bicycle was used by only 3.6% of the population and that cycling has increased unevenly on Italian territory. In Croatia, about 5% of all trips are made by bicycle and cycling is the least popular method of travel for going to work or to school (44). In Spain, only around 1.5% of children and 3% of adults cycle daily (45–48) and cycling is still classified as emerging (45). Portugal is the country in group 2 with the highest reported learning age. According to Shaw et al. (37), in Portugal bicycles play a scarce role on children's mobility—either alone or accompanied by parents—in terms of journeys between home and school. The reasons explaining the low levels of active and independent traveling in Portugal are related to stranger danger and the parents' perception of traffic hazards (49).

In countries from group 2, there have been efforts to promote sustainable mobility through the proliferation of soft modes of travel in urban centers and the planning of infrastructure to promote these behaviors (e.g., increasing bike lanes, bicycle share programs, etc.), and a number of cycling related community projects and initiatives have emerged [e.g., (50, 51)]. However, the majority of people opt for passive motor transport, with negative impact for health. Also, habits of using other modes of transport (i.e., passive motor transport) have a negative impact on bicycle use. Adults who use passive motor transport (i.e., car or motorbike) as the most frequent type of transport to commute are the most strongly opposed to cycling (52). Alternatively, leisure cyclists are likely to be commuter cyclists in the future (45, 52). So, increasing the cycling experience (in the leisure-time and/or sport activities) increases the valuation of attitudinal beliefs and decreases the barriers to cycling as a way of transportation.

Group 3 comprises the two Latin American countries in our study (Brazil and Mexico), in which children learned to cycle independently at a later age. Brazil and Mexico are the two only countries in our study that are not considered high-income countries according to the GDP data of the world bank (33). Possibly, buying a bicycle in these countries does not represent a priority in the families' domestic budget. In Brazil, until 1999, the use of bicycles was related to three aspects: (1) leisure, widespread in all socioeconomic levels; (2) competitive or non-competitive sporting activity; (3) toys, representing the development of motor skills and experiencing the first moments of freedom for children between 6 and 12 years old (53). In the last decade, there have been some changes to promote the use of bicycles (e.g., construction of bike lanes and implementation of bicycle sharing systems in large cities and in tourist cities). This has motivated families to encourage children to ride their bicycles before the age of 5/6; favoring the performance of leisure activities in the family, generating greater well-being and increased physical activity for all family members; in addition to the use of bicycles as a means of transport (urban mobility) by families of all socioeconomic levels (54).

In Mexico, the use of the bicycle does not seem to be popular or widespread. A recent study based on the national intercensal survey of 2015 (30), showed that although active transportation to school is fairly common among Mexican schoolchildren and adolescents, cycling is not (66.2% of the students walk to school whereas only 1.6% cycles). Moreover, when considering active and passive transportation modes, the bicycle falls into the last position in all states of Mexico.

### Learning Age Over Generations

There could be some important and universal elements affecting the learning age for cycling (e.g., cooperation for learning, time on task, prompt feedback, etc.) that can be applied around the globe in diverse populations. Generation X learnt to cycle later than generation Z, which could be due to less financial availability of previous generations to acquire bicycles and to less shared leisure time between parents and children of those generations. In fact, parents and relatives of people in generation X had limited leisure time and consequently less time to support children in learning this motor task. Changes in the number of children per family, in the cultural ideals of parenthood and of parental levels of supervision and engagement in play should also be considered (55). For instance, it has been shown that younger siblings usually learn to cycle earlier than older siblings and only children (56). Homes, families and schools are powerful environments for empowering cycling enculturation (45). Specifically, family can be the social group with the most positive influence on the decision to use the bicycle (52) and/or learn to cycle. To do so, riding a bike should not only be seen as a mode of transport, guided by adult rationality, but as a form of active play, leisure, recreation or sport, which lays the foundation of positive practical experiences (45). Most children learn to cycle with the help of their families. Although structured programs with cycling lessons exist, they seem to be more frequent for children with disabilities (57). However, for all children, learning is enhanced when (1) it is more like a team effort than a solo race, (2) frequent opportunities to perform and receive suggestions for improvement exist and/or (3) learners use realistic amounts of time for effective learning (58). When a child persists, he/she can learn how to cycle. However, in addition to persistence, when the child is supported by an adult who provides feedback and encouragement to help him/her learn how to cycle, the path of learning might increase. In addition, the design of bicycles and their size have become more child-friendly across generations. It is possible that many children from generation X did not have access to bicycles correctly body-scaled to them (59), and instead they had to use adult bicycles. After entering the new millennium, our findings reveal another drop in the age that children learn how to cycle independently. Possibly, this is due not only to the more central place the bike adopts within the family context nowadays, but also due to the transition from learning how to cycle with training wheels to the use of the balance bikes (55, 60). In many countries, cycling is considered a traditional leisure time activity during childhood as many parents offer bikes to their children for playing when they are young. Moreover, cycling has become increasingly valued for its potential to improve physical literacy/motor skills and physical activity (61). Also, the greater importance that has been given in the recent decades to policies that promote healthy lifestyles and active transport (62) is probably another factor that influences the cycling learning age.

The results of the present study reveal the influence of changes in the macrosystem and chronosystem dimensions in proximal processes related to learning how to cycle, between generations X and Z. These changes are essential for the child to be able to cycle in the public space, alone or with other children, without adult supervision. This is one of the main licenses of independent mobility and a privileged component in a child-friendly community (63).

As a way of low-carbon city strategies around the World, policy and decision makers are engaged in the planning of initiatives that promote cycling (46) and there has been increased attention for cycling and its infrastructure to meet climate goals (64).

### Learning Age Over Generations by Country

The bike is increasingly being considered an enjoyable, cheap, ecological and efficient mode of transportation, which promotes health and well-being. During youth, appropriate cycling promotion is associated with empowering confidence, knowledge, competence and attitudes (65).

Our findings revealed a significant interaction between country and generation indicating that generational changes differed slightly among countries with the decline in learning age being less evident in countries in which the learning age has been consistently low across generations.

It is not surprising that in countries from group 1 (especially in the Netherlands), the learning age has not changed as much over generations as in countries from group 2 or 3, in which investments in infra-structures and policies that promote cycling are more recent. However, it should be noted that the interaction effect was negligible ($\eta_{\text{p}}^{2}$ = 0.006). Whilst learning age for independent cycling is generally lower in later generations, countries generally maintained their rank relative to one another across time. Although national governments have increased their support for cycling in the past decades, there are other factors that may hinder or facilitate cycling. For instance, the perception of road safety has changed across generations, and fears about safety have often been mentioned as a reason to not cycle, especially in countries from group 2 (e.g., UK, see 18) and 3 (e.g., Mexico, see 30). The cycling culture and the efficacy of cycling campaigns also vary, and the plans for cycling investment are diverse (66). It is thus clear that policies and an urban planning framework that is friendly to the use of bicycle as an expressive daily travel mode by children and adults, as well as valuing the learning of cycling as an educational and developmental asset, are paramount for a widespread cycling culture and behavior.

### Strengths and Limitations

To our knowledge, this is the first international study that examines the age for learning to cycle in different countries and across time. Additionally, this study has a large sample size, which was made possible through a web-based survey. However, some limitations need to be considered when interpreting the present findings. First, this cross-sectional study used retrospective self-report and parental reports, which are prone to response bias. Future studies should involve longitudinal designs to further understand when and how (young) children learn to cycle. Second, as participant recruitment took place *via* social media and with the help of different partners (e.g., parents' magazines, early childhood teachers' organizations, cycling federations and non-profit organizations), this may have led to sampling bias. For instance, members of cycling organizations will cycle more than the general populations in the respective countries, which might bias the average learning age for cycling within the study sample. Further research should consider personal/demographic factors (e.g., socio-economic status, ethnicity) and psychosocial factors (e.g., attitudes, habits, perceptions) to gain better insights into learning to cycle (67). Third, there were some large differences in sample size and variance between countries although unequal N HSD *post-hoc* tests have been used to address the issue of unequal group size and heterogeneity of variance. Finally, the present study focused on comparing learning ages for cycling among but not within countries. As cycling levels have been shown to vary within countries (68, 69), future studies should explore potential differences in learning age within countries and regions.

## Conclusions

Learning to cycle is associated with physical, social and emotional benefits; as such, the earlier a child can ride a bike independently, the sooner they will experience those benefits. The present study specifically examined the influence of the macrosystem and chronosystem-as noted in Bronfenbrenner's bioecological theory (1995)-and their interaction, on the learning age of cycling. The interaction effect between country and generation was negligible, but our results show that the difference in age for learning to cycle independently can vary by about 3 years (usually between 4 and 7 years of age), depending on the country where the child lives (macrosystem) and the time of birth (chronosystem). A child from generation Z born in a high-income country where the bicycle use is culturally imbedded (e.g., the Netherlands or Finland), will on average learn to cycle independently at a much younger age than a child from generation X born in a middle-income country without a strong cycling culture (e.g., Brazil or Mexico). This latter group of countries would benefit the most from cycling promotion campaigns, policies that improve perceived public and road safety, and investments in infrastructures to promote active modes of transport. Finally, learning to cycle depends mostly on the child's microsystem—specifically, the face-to-face interactions between the children and the adults that support them to cycle. Our results indicate that the cycling culture, expressed by the cycling modal share that exists in a given country and a given time, seems to be closely related to the age at which children learn how to cycle, highlighting the interdependency between the different levels of a person's environment (19, 20).

## Data Availability Statement

The raw data supporting the conclusions of this article will be made available by the authors, without undue reservation.

## Ethics Statement

The studies involving human participants were reviewed and approved by the Ethics Committee of the Faculty of Human Kinetics (process number 22/2019). The patients/participants provided their written informed consent to participate in this study.

## Author Contributions

RC, CM, MB, FL, and DC conceived the project and tested the first version of the survey. RC was responsible for a first data analysis and a first draft of the manuscript, but all authors contributed for the analysis and writing process and identified and recruited the international partners. All international partners (EH, AL, PT, GF, CS, BJ, LZ, AD, FB, RF, SV, SZ, and IE) examined the survey, reached a consensus for the final version, and were responsible for translating the survey and testing it in their own countries. All authors contributed to the dissemination of the survey in their own countries and read and approved the final manuscript.

## Funding

RC, CM, and MB were partly supported by the Portuguese Foundation for Science and Technology, under Grant UIDB/00447/2020 to CIPER—Centro Interdisciplinar para o Estudo da Performance Humana (unit 447); DC was partly supported by the Portuguese Foundation for Science and Technology, under project No. UID/CED/04748/2020.

## Conflict of Interest

The authors declare that the research was conducted in the absence of any commercial or financial relationships that could be construed as a potential conflict of interest.

## Publisher's Note

All claims expressed in this article are solely those of the authors and do not necessarily represent those of their affiliated organizations, or those of the publisher, the editors and the reviewers. Any product that may be evaluated in this article, or claim that may be made by its manufacturer, is not guaranteed or endorsed by the publisher.

## References

[1] Zeuwts L Ducheyne F Vansteenkiste P D'Hondt E Cardon G Lenoir M. Associations between cycling skill, general motor competence and body mass index in 9-year-old children. Ergonomics. (2015) 58:160–71. 10.1080/00140139.2014.961971 25351715

[2] Haubenstricker J Seefeldt V. Acquisition of motor skills during childhood. In: Seefeldt V editor. Physical Activity and Well-being. Waldorf, MD.: American Alliance for Health, Physical Education, Recreation and Dance Publications (1986). p. 41–92.

[3] Malone K Rudner J. Global perspectives on children's independent mobility: a socio-cultural comparison and theoretical discussion of children's lives in four countries in Asia and Africa. Glob Stud Child. (2011) 1:243–59. 10.2304/gsch.2011.1.3.243

[4] Marzi I Reimers AK. Children's independent mobility: current knowledge, future directions, and public health implications. Int J Environ Res Public Health. (2018) 15:2441. 10.3390/ijerph15112441 30388880 PMC6267483

[5] Ikeda E Hinckson E Witten K Smith M. Assessment of direct and indirect associations between children active school travel and environmental, household and child factors using structural equation modelling. Int J Behav Nutr Phys Act. (2019) 16:32. 10.1186/s12966-019-0794-5 30953526 PMC6451289

[6] Fyhri A Hjorthol R. Children's independent mobility to school, friends and leisure activities. J Transp Geogr. (2009) 17:377–84. 10.1016/j.jtrangeo.2008.10.010

[7] Smith M Hosking J Woodward A Witten K MacMillan A Field A. Systematic literature review of built environment effects on physical activity and active transport-an update and new findings on health equity. Int J Behav Nutr Phys Act. (2017) 14:158. 10.1186/s12966-017-0613-9 29145884 PMC5693449

[8] Hulteen RM Morgan PJ Barnett LM Stodden DF Lubans DR. Development of foundational movement skills: a conceptual model for physical activity across the lifespan. Sports Med. (2018) 48:1533–40. 10.1007/s40279-018-0892-6 29524160

[9] Oja P Titze S Bauman A de Geus B Krenn P Reger-Nash B. Health benefits of cycling: a systematic review. Scand J Med Sci Sports. (2011) 21:496–509. 10.1111/j.1600-0838.2011.01299.x 21496106

[10] Neun M Haubold H. The EU Cycling Economy–Arguments for an integrated EU cycling policy. Brussels (2016).

[11] Heinen E van Wee B Maat K. Commuting by bicycle: an overview of the literature. Transp Rev. (2010) 30:59–96. 10.1080/01441640903187001

[12] Pucher J Buehler R. Cycling towards a more sustainable transport future. Transp Rev. (2017) 37:689–94. 10.1080/01441647.2017.1340234

[13] Larouche R Saunders TJ Faulkner G Colley R Tremblay M. Associations between active school transport and physical activity, body composition, and cardiovascular fitness: a systematic review of 68 studies. J Phys Act Health. (2014) 11:206–27. 10.1123/jpah.2011-0345 23250273

[14] United Nations. Transforming our World: The 2030 Agenda for Sustainable Development A/RES/70/01. United Nations (2015).

[15] Agervig Carstensen T Ebert A-K. Chapter 2 cycling cultures in northern Europe: from ‘golden age' to ‘renaissance’. In: John P editor. Cycling and Sustainability. Transport and Sustainability. 1: Emerald Group Publishing Limited (2012). p. 23–58. 10.1108/S2044-9941(2012)0000001004

[16] Tschoerner-Budde C. Cycling policy futures: diversifying governance, expertise and the culture of everyday mobilities. Applied Mobilities. (2020) 5:306–23. 10.1080/23800127.2020.1766217

[17] Watson M. How theories of practice can inform transition to a decarbonised transport system. J Transp Geogr. (2012) 24:488–96. 10.1016/j.jtrangeo.2012.04.002

[18] Golbuff L Aldred R. Cycling Policy in the UK: a Historical and Thematic Overview. London: University of East London Sustainable Mobilities Research Group (2011).

[19] Bronfenbrenner U. Developmental ecology through space and time: A future perspective. In: Moen P Elder GH Jr Luscher K editors. Examining Lives in Context: Perspectives on the Ecology of Human Development. Washington, DC: American Psychological Association (1995). p. 619–47. 10.1037/10176-018

[20] Bronfenbrenner U Morris PA. The bioecological model of human development. In Lerner RM Damon W editors. Handbook of Child Psychology. Hoboken, NJ: Wiley (2006). p. 793–828.

[21] Temple VA Purves PL Misovic R Lewis CJ DeBoer C. Barriers and Facilitators for Generalizing Cycling Skills Learned at Camp to Home. Adapt Phys Activ Q. (2016) 33:48–65. 10.1123/APAQ.2015-0040 26785500

[22] de Vries SI Hopman-Rock M Bakker I Hirasing RA van Mechelen W. Built environmental correlates of walking and cycling in Dutch urban children: results from the SPACE study. Int J Environ Res Public Health. (2010) 7:2309–24. 10.3390/ijerph7052309 20623026 PMC2898051

[23] Oosterhuis H. Cycling, modernity and national culture. Soc Hist. (2016) 41:233–48. 10.1080/03071022.2016.1180897

[24] Cox P. CYCLING: a sociology of velomobility. Sl: ROUTLEDGE (2020). 10.4324/9781315533698

[25] Haustein S Koglin T Nielsen TAS Svensson Å. A comparison of cycling cultures in Stockholm and Copenhagen. Int J Sustain Transp. (2020) 14:280–93. 10.1080/15568318.2018.1547463

[26] Goel R Goodman A Aldred R Nakamura R Tatah L Garcia LMT. Cycling behaviour in 17 countries across 6 continents: levels of cycling, who cycles, for what purpose, and how far? Transp Rev. 2021:1–24. 10.1080/01441647.2021.1915898

[27] Withagen R de Poel HJ Araújo D Pepping G-J. Affordances can invite behavior: Reconsidering the relationship between affordances and agency. New Ideas Psychol. (2012) 30:250–8. 10.1016/j.newideapsych.2011.12.003

[28] European Cyclists' Federation. Cycling Data Map. Brussels (2021).

[29] Cycling UK. Cycling UK's Cycling Statistics (2021). Available online at: https://www.cyclinguk.org/statistics.

[30] Ortiz-Hernández L Vega-López A Ayala-Hilario C. Factores sociodemográficos asociados con los modos de transporte en escolares y adolescentes mexicanos [Commuting to school among Mexican schoolchildren and adolescents]. Boletí*n Médico del Hospital Infantil de México*. (2019) 76:225–36. 10.24875/BMHIM.19000161 31536044

[31] Reibold A. Advocacy Success: New Finnish Government to Increase Cycling by 30%: European Cyclists Federation (2019). Available online at: https://ecf.com/news-and-events/news/advocacy-success-new-finnish-government-increase-cycling-30.

[32] Government roadmap to carbon neutral Finland - climate leadership means opportunities for the whole country [press release]. Helsinki: Finnish Government (2020).

[33] GDP per capita, PPP. International Comparison Program, World Bank | World Development Indicators database, World Bank | Eurostat-OECD PPP Programme (2021). Available online at: https://data.worldbank.org/indicator/NY.GDP.PCAP.PP.CD.

[34] Tremblay MS Barnes JD González SA Katzmarzyk PT Onywera VO Reilly JJ. Global Matrix 2. 0: report card grades on the physical activity of children and youth comparing 38 countries. J Phys Act Health. (2016) 13:S343. 10.1123/jpah.2016-0594 27848745

[35] Aubert S Barnes JD Abdeta C Abi Nader P Adeniyi AF Aguilar-Farias N. Global Matrix 3. 0 physical activity report card grades for children and youth: results and analysis from 49 countries. J Phys Act Health. (2018) 15:S251–73. 30475137 10.1123/jpah.2018-0472

[36] Hanenbergh K Röben M. Ons Stalen Ros, Nederland wordt een land van fietsers 1820 tot 1920. Utrecht: De Vrije Uitgevers (2015).

[37] Shaw B Bicket M Elliott B Fagan-Watson B Mocca E Hillman M. Children's independent mobility. An International Comparison and Recommendations for Action London. London: Policy Studies Institute (2015).

[38] Larouche R Garriguet D Tremblay M. Outdoor time, physical activity and sedentary time among young children: The 2012-2013 Canadian Health Measures Survey. Can J Public Health. (2017) 107:e500–e6. 10.17269/CJPH.107.5700 28252366 PMC6972245

[39] Holfelder B Schott N. Relationship of fundamental movement skills and physical activity in children and adolescents: a systematic review. Psychol Sport Exerc. (2014) 15:382–91. 10.1016/j.psychsport.2014.03.005

[40] Sääkslahti A Niemistö D. Outdoor activities and motor development in 2–7-year-old boys and girls. J Phys Educ Sport. (2021) 21:463–8. 10.7752/jpes.2021.s1047

[41] Ducheyne F De Bourdeaudhuij I Lenoir M Spittaels H Cardon G. Children's cycling skills: Development of a test and determination of individual and environmental correlates. Accid Anal Prev. (2013) 50:688–97. 10.1016/j.aap.2012.06.021 22795546

[42] Zeuwts L Vansteenkiste P Cardon G Lenoir M. Development of cycling skills in 7- to 12-year-old children. Traffic Inj Prev. (2016) 17:736–42. 10.1080/15389588.2016.1143553 26889690

[43] Legambiente. L'A BI CI 2 Rapporto sull'economia della bicicletta in Italia 2018 (2018). Available online at: https://www.legambiente.it/sites/default/files/docs/rapporto_economia_bicicletta_labici_2018.pdf.

[44] Republic of Croatia. Transport Development Strategy of the Republic of Croatia (2017–2030). Zagreb (2017).

[45] Jordi-Sánchez M. Social perceptions of the promotion of cycling as a mode of transport for children in Andalusia (Spain). J Transp Geogr. (2018) 72:86–93. 10.1016/j.jtrangeo.2018.08.014

[46] Braun LM Rodriguez DA Cole-Hunter T Ambros A Donaire-Gonzalez D Jerrett M. Short-term planning and policy interventions to promote cycling in urban centers: Findings from a commute mode choice analysis in Barcelona, Spain. Transp Res A: Policy Pract. (2016) 89:164–83. 10.1016/j.tra.2016.05.007

[47] Plasencia-Lozano P. Evaluation of a new urban cycling infrastructure in caceres (Spain). Sustainability. (2021) 13:1910. 10.3390/su13041910

[48] Estevan I Queralt A Molina-Garcia J. Biking to school: the role of bicycle-sharing programs in adolescents. J Sch Health. (2018) 88:871–6. 10.1111/josh.12697 30392192

[49] Lopes F Cordovil R Neto C. Children's independent mobility in Portugal: effects of urbanization degree and motorized modes of travel. J Transp Geogr. (2014) 41:210–9. 10.1016/j.jtrangeo.2014.10.002

[50] Municipality of Lisbon. Programa Municipal de Comboios de Bicicletas de Lisboa [Lisbon Municipal Bicycle Train Program]. (2021). Available online at: https://www.lisboa.pt/cidade/mobilidade/escolar/comboios-de-bicicleta.

[51] MUBI. Envolve-te nos Orçamentos Participativos da tua cidade [Get involved in the Participatory Budgets of your city] (2021). Available online at: https://mubi.pt/2021/05/25/envolve-te-nos-orcamentos-participativos-da-tua-cidade/.

[52] Muñoz B Monzon A Lois D. Cycling habits and other psychological variables affecting commuting by bicycle in Madrid, Spain. Transp Res Rec. (2013) 2382:1–9. 10.3141/2382-01

[53] Ritta LAS. Motivos de uso e não-uso de bicicletas em Porto Alegre: um estudo descritivo com estudantes da UFRGS [Reasons for using and not using bicycles in Porto Alegre: a descriptive study with UFRGS students] 2012.

[54] Duran AC Anaya-Boig E Shake JD Garcia LMT Rezende LFMd Hérick de Sá T. Bicycle-sharing system socio-spatial inequalities in Brazil. J Transp Health. (2018) 8:262–70. 10.1016/j.jth.2017.12.011

[55] Laukkanen A Hasanen E Matilainen P. Pyöräilytaidon oppimista selittävät yksilö-, ympäristö- ja tehtävätason tekijät 1950–2010-luvuilla. Liikunta & Tiede. (2021) 58:91–8. Available online at: https://www.lts.fi/media/lts_vertaisarvioidut_tutkimusartikkelit/2021/lt_4_2021-91-98.pdf

[56] Mercê C Branco M Catela D Lopes F Rodrigues LP Cordovil R. Learning to Cycle: Are Physical Activity and Birth Order Related to the Age of Learning How to Ride a Bicycle? Children. (2021) 8:487. 10.3390/children8060487 34200996 PMC8226627

[57] Mercê C Pereira JV Branco M Catela D Cordovil R. Training programmes to learn how to ride a bicycle independently for children and youths: a systematic review. Phys Educ Sport Pedagogy. 2021:1–16. 10.1080/17408989.2021.2005014

[58] Chickering AW Gamson ZF. Seven principles for good practice in undergraduate education. AAHE bulletin. (1987) 3:7.

[59] Fajen BR Riley MA Turvey MT. Information, affordances, and the control of action in sport. Int J Sport Psychol. (2009) 40:79–107.

[60] Mercê C Branco M Catela D Lopes F Cordovil R. Learning to cycle: from training wheels to balance bike. Int J Environ Res Public Health. (2022) 19:1814. 10.3390/ijerph19031814 35162834 PMC8834827

[61] Hulteen RM Smith JJ Morgan PJ Barnett LM Hallal PC Colyvas K. Global participation in sport and leisure-time physical activities: a systematic review and meta-analysis. Prev Med. (2017) 95:14–25. 10.1016/j.ypmed.2016.11.027 27939265

[62] European Cyclists' Federation. The benefits of cycling: Unlocking their potential for Europe. (2018). Available online at: https://ecf.com/sites/ecf.com/files/TheBenefitsOfCycling_final-v2.pdf.

[63] Lopes F Cordovil R Neto C. Independent mobility and social affordances of places for urban neighborhoods: a youth-friendly perspective. Front Psychol. (2018) 9:2198. 10.3389/fpsyg.2018.02198 30483200 PMC6243082

[64] Federal Ministry Republic of Austria. Pan-European Master Plan for Cycling Promotion (2021). Available online at: https://thepep.unece.org/sites/default/files/2021-05/MASTERPLAN_2021-05-16_BF.pdf.

[65] Sersli S DeVries D Gislason M Scott N Winters M. Changes in bicycling frequency in children and adults after bicycle skills training: a scoping review. Transp Res A: Policy Pract. (2019) 123:170–87. 10.1016/j.tra.2018.07.012

[66] Colli E. New analysis: Cycling earns its place in COVID-19 recovery plans (2021). Available from: https://ecf.com/news-and-events/news/new-analysis-cycling-earns-its-place-covid-19-recovery-plans (accessed May 31, 2021).

[67] Willis DP Manaugh K El-Geneidy A. Cycling under influence: summarizing the influence of perceptions, attitudes, habits, and social environments on cycling for transportation. Int J Sustain Transp. (2015) 9:565–79. 10.1080/15568318.2013.827285

[68] Buhler R Pucher J. International overview of cycling. In: Puhler RBJ editor. Cycling for sustainable cities. Cambridge, MA: The MIT press (2021). p. 11–34. 10.7551/mitpress/11963.003.0006

[69] Goodman A Aldred R. Inequalities in utility and leisure cycling in England, and variation by local cycling prevalence. Transp Res F: Traffic Psychol Behav. (2018) 56:381–91. 10.1016/j.trf.2018.05.001

